# A Knowledge Transfer Framework for General Alloy Materials Properties Prediction

**DOI:** 10.3390/ma15217442

**Published:** 2022-10-24

**Authors:** Hang Sun, Heye Zhang, Guangli Ren, Chao Zhang

**Affiliations:** 1 School of Biomedical Engineering, Shenzhen Campus of Sun Yat-sen University, Shenzhen 518107, China; 2 Department of Pediatric, General Hospital of Southern Theater Command of PLA, Guangzhou 510010, China

**Keywords:** alloy, property prediction, data process, machine learning, transfer learning

## Abstract

Biomedical metal implants have many applications in clinical treatment. Due to a variety of application requirements, alloy materials with specific properties are being designed continuously. The traditional alloy properties testing experiment is faced with high-cost and time-consuming challenges. Machine learning can accurately predict the properties of materials at a lower cost. However, the predicted performance is limited by the material dataset. We propose a calculation framework of alloy properties based on knowledge transfer. The purpose of the framework is to improve the prediction performance of machine learning models on material datasets. In addition to assembling the experiment dataset, the simulation dataset is also generated manually in the proposed framework. Domain knowledge is extracted from the simulation data and transferred to help train experiment data by the framework. The high accuracy of the simulation data (above 0.9) shows that the framework can effectively extract domain knowledge. With domain knowledge, the prediction performance of experimental data can reach more than 0.8. And it is 10% higher than the traditional machine learning method. The explanatory ability of the model is enhanced with the help of domain knowledge. In addition, five tasks are applied to show the framework is a general method.

## 1. Introduction

**Figure 1:**
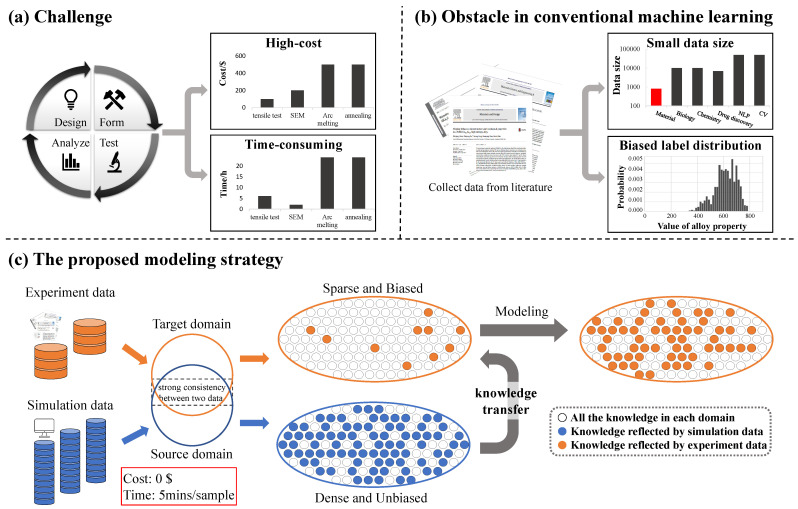
Strategy for alloy material properties prediction in this work. (**a**) Measuring the properties of alloy materials through traditional material experiments will face the challenge of high cost and high time consumption. (**b**) The data used by conventional machine learning methods for material properties prediction often suffer from the obstacles of small data size and biased label distribution. (**c**) In this work, we propose a machine learning modeling strategy that uses knowledge transfer to solve the challenges of predicting alloy properties and the obstacles of conventional machine learning methods.

The reliable prediction of material properties can accelerate the design of new alloy materials. As a common biological material, metal alloys have been widely used in medical fields such as medical implants and medical devices [1,2]. In addition to existing applications, there are still numerous special medical scenes that potentially require the participation of alloy biomaterials. To meet the application of special scenes, it is necessary to design new alloy materials with appropriate properties. However, it is complex to develop alloy materials with appropriate material properties according to the design requirements. The properties of alloy materials are affected by many factors. Composition and processing are the main factors of material properties. Adding new elements or adjusting the element content ratio may bring significant changes to the properties of the alloy. The alloys with the same composition will show different material properties after different treatment processes. A variety of candidate alloys that need to measure the properties of materials will be produced in the design process. Therefore, measuring the alloy properties is an important task. However, the traditional method of measuring alloy material properties is still complicated. As shown in Figure 1a, the properties of an alloy candidate can be obtained through four steps: design, forming, testing, and analysis. A variety of material experiments should be carried out in the forming steps and testing steps. In addition, it takes several cycles to complete the development of new alloy material. Traditional methods face the challenge of high-cost and time-consuming [3,4]. The price of the experimental equipment is shown in Figure 1a, and the time of the corresponding experiment is investigated from the website www.made-in-china.com. It is a popular subject to find an effective method to predict the properties of alloy materials.

Machine learning is an effective method for predicting the properties of new materials. With the development of computing science, there have been a large number of examples of solving problems in the biology and medicine field with the help of computers. The prediction of alloy material properties is also one of the topics promoted by computational science. Combined with model-based theoretical science a few centuries ago, material properties can be predicted in the form of mathematical equations. Simulation methods such as density functional theory (DFT) and molecular dynamics (MD) appeared with the help of computers. However, the high computational cost limits the implementation of the above two traditional prediction methods. Different from the traditional prediction methods, summarizing the law of experimental data is another effective pattern for establishing accurate prediction methods. The experiment data directly reflect the chemical and physical phenomena of the material. Machine learning can learn the laws contained in a dataset. Plenty of current machine learning related innovations in the material field have successfully accelerated material discovery [5,6,7,8]. In recent years, various types of properties of alloys can be predicted by machine learning methods. Heat treatment process performance, mechanical properties, plastic and superplastic behaviors, and other material properties can be accurately predicted by machine learning methods [9,10,11,12]. However, as shown in Figure 1b, the obstacles are also apparent when designing alloys with machine learning. A common obstacle in material science is that materials datasets are typically small compared to other fields [13,14,15]. It is difficult to collect a large number of experimental material data due to the high cost of material experiments. Another obstacle is that the label distribution in the material dataset is usually biased [16]. Data in material datasets are usually collected from published literature and public database. Few failed experimental samples are included in the datasets. As a result, the material dataset lacks a lot of valuable information. These obstacles in the material datasets limit the prediction of alloy material properties by machine learning methods.

It is practically challenging to design a general prediction method applying to a huge amount of alloy candidates. As mentioned above, the obstacles in material datasets limit the predictive ability of machine learning models. The alloy candidate space is much larger than the range of the data set. To make the machine learning model can be applied in the alloy candidate space, there is an urgent need for the machine learning model to have the ability to extrapolate. Coupling domain knowledge with a machine learning model is an effective strategy for building models with extrapolation [17,18]. Feature engineering is a common method to establish domain knowledge coupling machine learning models. However, complex descriptors introduced by feature engineering make the application of the model more difficult. As an important branch of machine learning, transfer learning focuses on applying the gained knowledge to a related problem [19]. As a mature strategy, transfer learning has been applied in many fields [20,21]. The deep neural network is a flexible method that has the advantage of exploiting transfer learning [22,23]. We attempt to establish the domain knowledge coupling deep neural networks based on the transfer learning strategy.

In this work, we propose a highly general alloy material properties prediction framework based on transfer learning. Through this framework, a machine-learning model reflecting the relationship between alloy composition and property can be established. The strategy of the framework is shown in Figure 1c. Distinct from previous works, we aim to bring domain knowledge to machine learning models without using complex descriptors. The source of domain knowledge is the mainstream alloy properties simulation software Jmatpro and Thermo-calc. Simulation software calculates the properties of the material through embedded empirical formula [24]. The formula is a reliable source of knowledge because it has been examined for a long time in the materials field. The proposed framework is to utilize the domain knowledge extracted from simulation software to assist in the training of experiment data. Simulation data and experimental data converge. The experimental data can be trained faster and more accurately because of the domain knowledge provided by the simulation data. Fine-tuning the experimental data after pre-training of the simulation data makes up for the error of the formula in the simulation software. Because the experimental data are set to ground truth, the framework can achieve higher accuracy than the simulation software. To prove the generality, we apply the proposed framework to five independent predictions of alloy properties: time-temperature-transition diagram (TTT), martensite initial temperature (Ms), Young’s modulus (E), continuous cooling transition diagram (CCT), and phase diagram (P). The five tasks were selected on the basis of covering a number of sub-areas, including phase diagrams, mechanical properties, and heat treatment performance prediction. And their simulation data can be generated in the above simulation software. In addition, the proposed framework can also predict the properties of new materials, which only uses experimental data. With the help of other relevant domain knowledge, performance can also be improved.

## 2. Materials and Methods

### 2.1. Datasets

In this article, the proposed framework is applied to five tasks to demonstrate the generality of our framework. Each task contains two types of datasets: the simulation dataset and the experiment dataset. The simulation datasets are collected by alloy properties simulation software. The experiment datasets are compiled from the available published literature. The five tasks are the prediction of the time-temperature-transition diagram (TTT), martensite initial temperature (Ms), Young’s modulus (E), continuous cooling transformation diagram (CCT), and phase diagram (P), respectively.

#### 2.1.1. TTT

The TTT diagram consists of multiple C-shaped curves. Each curve represents the time and temperature of the start or end of each alloy phase. The specific temperature and time are respectively shown by the horizontal and vertical coordinates of the diagram. Since the TTT graph is two-dimensional data, we treat it as a one-dimensional form. The curve is intercepted into multiple points. Each point is used as a training sample. We take the pearlite starting curve as the representative of this task. The TTT task is set as a regression task. The inputs are the composition of the alloy and the time value of each point. The output is the temperature value corresponding to that point. The experimental data of the TTT task come from literature [9].

#### 2.1.2. Ms

The Ms task is set as a regression task to predict the martensite transformation temperature of the alloy. The Ms dataset is composed of the martensite transformation temperature of a variety of alloys treated at different austenitization temperatures. The sample in the Ms dataset is one-dimensional data, which records the content of each element of the alloy, austenitization temperature, and the corresponding Ms temperature. The inputs of the Ms dataset are the composition and austenitization temperature. The output is the martensite transformation start temperature. The experimental data of Ms task come from literature [17].

#### 2.1.3. E

The E task is set as a regression task to predict Young’s modulus of the alloy. The E dataset consists of Young’s modulus of various alloys at different temperatures. The sample in the E dataset is one-dimensional data, which records the content of each element of the alloy, temperature, and the corresponding Young’s modulus. The inputs of the Ms dataset are the composition and temperature. The output is the value of Young’s modulus. The experimental data of E task come from literature [25].

#### 2.1.4. CCT

The format of the CCT diagram is similar to the TTT diagram. The CCT diagram also contains multiple curves, representing the start or end of each alloy phase. The data representation is the same as the TTT diagram, the curve is divided into multiple points as separate samples. The bainite starting curve is selected as the representative. The inputs are the composition of the alloy and temperature at one point, and the output is the time at the point. The experimental data of the CCT task come from literature [26].

#### 2.1.5. Phase

The phase diagram prediction task is set as a binary classification problem. The phase dataset is composed of alloy phases of different alloys at different temperatures. Single-phase solid solution alloys are classified as class SS, and multiphase alloy systems with a mixture of solid solution and intermetallic are classified as class SS + IM. The inputs of the phase dataset are the composition and temperature. The output is the classification of alloy phases. The experimental data of Phase task come from literature [27].

### 2.2. Simulation Software

The thermodynamics knowledge combined with machine learning is derived from the commercial simulation software JMatPro and Thermo-calc. In this section, we introduce the process of using Jmatpro and Thermo-Calc tool databases to generate large simulation data sets of multiple composition alloy properties data. The working principle of two software for calculating and generating simulation data is also introduced. The scientific principles of simulation software can be reflected when there is a sufficient amount of data. To make the simulation dataset fully reflect the thermodynamic knowledge of Thermo-calc, we collect as much simulation data as possible to increase the coverage of the dataset. The content of each element ranges from 0 to the maximum value that can be calculated by the software. The contents of various elements are mostly distributed near the common values. There is also a small amount of extreme content value data to ensure that there is knowledge in extreme cases in the data set. Then the randomly initialized deep neural network (source model) is used to extract knowledge from the simulation data. The training effect of the source model is taken as the index to verify the extraction of thermodynamic knowledge.

#### 2.2.1. Jmatpro

The Jmatpro software generates four simulation data sets, which are TTT, Ms, CCT, and E. Jmatpro is used to calculate a wide range of properties of multicomponent alloy materials in industrial practice. A variety of properties, including phase diagram transformation, and mechanical properties, can be calculated quickly and steadily by simple operation with Jmatpro. The TTT diagrams calculation model in Jmatpro is based on the Kirkaldy model. Kirkaldy model considers the grain size, Ae3 temperature, and effective diffusion coefficient to produce the ‘C’ curve using the general formula for the time to transform x fraction of austenite at a temperature T. And modifications were implemented after many verification experiments and theoretical derivation. The CCT diagrams are converted from the corresponding calculated TTT diagram using well-established additivity rules [28,29,30]. The Ms calculation model in Jmatpro incorporates certain important features of a full thermodynamic treatment, each element contributes to the stability of both the parent and the product phases. By combining appropriate mathematical functions, the model automatically calculates the Ms in different environments from the alloy composition [31].

#### 2.2.2. Thermo-Calc

The Thermo-calc software generates three simulation data sets, which are TTT, CCT, and Phase. The function of phase diagram calculation is implemented by the CALPHAD method embedded in Thermo-calc. The CALPHAD method is a semi-empirical method to mathematically describe the thermodynamic properties of multicomponent systems. It establishes a robust set of thermodynamic parameters based on a large amount of reliable experiment data and then implements the extrapolation of the unexperimental area after mathematical expression. The simulation data generated by Thermo-calc contain the above scientific principles [32].

### 2.3. Framework

**Figure 2:**
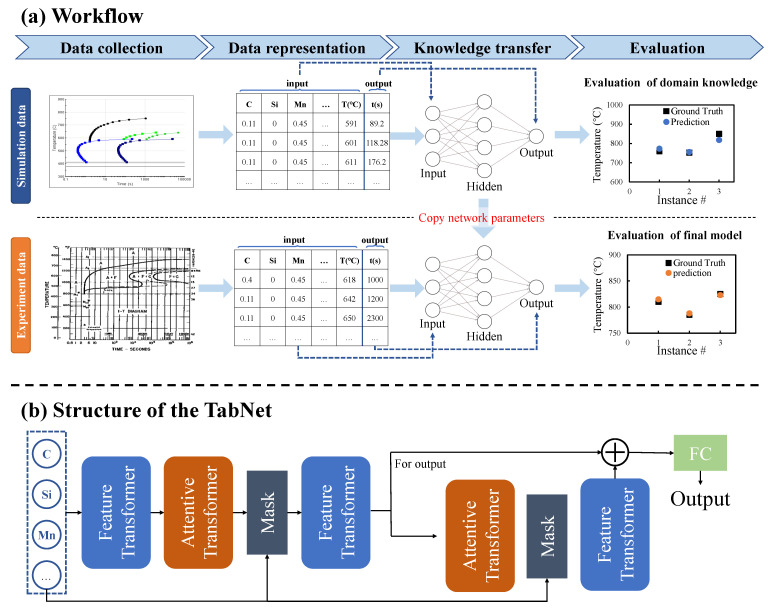
The workflow of the proposed framework. (**a**) The workflow in turn includes data collection, data representation, knowledge extraction, and knowledge transfer. Two types of datasets are collected: a large-scale simulation dataset from simulation software and a regular-sized experiment dataset from published literature. After data representation, the simulation dataset is trained first, and then the learned knowledge is transferred to the training of the experiment dataset by sharing model parameters. (**b**) The architecture schematic of deep neural network.

The main work of this paper is to devise a framework to predict alloy material properties. The procedure schematic of the framework is shown in Figure 2a. The first step of the framework is data collection. For one task, we collect corresponding simulation data and experimental data, respectively. Simulation data is generated from the simulation software. Experiment data is collected from published literature. Next, to unify the data format, we have to implement data representation to two kinds of raw data separately. The tasks presented in this work are displayed in different forms, including 1-D data and 2-D curve picture data. All data are uniformly represented in 1-D format with multiple input variables corresponding to one output variable. 1-D data takes properties as output and other elements of alloy as inputs. 2-D data is split into multiple points, each point is used as a single sample, one coordinate of the point is used as the output, and the other coordinate together with other elements is used as the input. The specific data representation operations for each task are described in the **Materials and Methods** section. After data representation, the simulation dataset of each task will be fed to the randomly initialized deep neural network. The deep neural network established after training is called the source model in transfer learning. The purpose of establishing the source model is to use the data-fitting function of the neural network to extract the domain knowledge from the simulation software. Although the network does not directly contain the laws of domain knowledge, It can imitate this knowledge and calculate similar outputs based on inputs. At the knowledge transfer stage, we obtain a machine learning model that extracts the thermodynamic knowledge from the simulation software. Then transfer learning is to fine-tune the model with the experiment data. At the fine-tuning stage, experiment data are fed into the target model which is a manually initialized deep neural network with the same structure as the source model. The initialization parameters of the target model are copied from the source model. The training effect of the final target model is evaluated based on experimental data.

The machine learning model is selected as a deep neural network because of its advantage in exploiting transfer learning. The deep neural network can be easily reused in related tasks, which fits well with transfer learning strategies. The model used in the proposed framework is TabNet, the structure is shown in Figure 2b [33]. The TabNet network is mainly composed of a Feature transformer layer, an Attentive transformer layer, and a mask layer.

(1) Feature transformer layer: Feature calculation, split for the decision step output, and information for the subsequent step. It can be seen that the Feature transformer layer consists of two parts. The parameters of the first half of the layer are shared, which means that they are jointly trained on all steps; while the second half is not shared, and is trained separately on each step. For each step, the input is the same features, so we can use the same layer to do the common part of the feature calculation, and then use different layers to do the feature part of each step. GLU is a gated linear unit, which is based on the original FC layer plus gating. The residual connection is used in the layer, and it is multiplied by $\sqrt{2}$ to ensure the stability of the network. The Feature transformer layer realizes the calculation of the features selected in the current step.
(2) Attentive transformer layer: feature selection, the function of this layer is to calculate the Mask layer of the current step based on the result of the previous step. A learnable mask layer is used to select salient features, and through the selection of sparse features, the learning of the model is more effective in each step. If a feature has been used many times in the previous step, it should no longer be selected by the model. Therefore, the model uses this Prior scales item to reduce the weight ratio of this type of feature. The Attentive transformer layer can obtain the Mask matrix of the current step according to the results of the previous step, and try to make the Mask matrix sparse and non-repetitive. The Mask vector of different samples can be different, which means that TabNet can allow different samples to choose different features instance-wise, and this feature is not available in tree models. For additive models such as XGBoost, a step is a tree. And the features used in this decision tree are selected on all samples, it cannot be instance-wise.

In general, TabNet uses a sequential multi-step framework to construct a neural network similar to an additive model. The key points in the model are the Attentive transformer layer and the Feature transformer layer.

### 2.4. Experimental Tools and Setup

The algorithm part of the proposed framework is implemented using Python. The model used in the framework is the deep neural network. The deep neural network is built with the help of the PyTorch Library. The input and output of the deep neural network are shown in the datasets section. For each dataset, we randomly split the data into 90%–10% train-test sets. 10% of the training set is split into validation sets. The model fitting is based on the accuracy of the validation set. In transfer learning, the source model is trained on the training set of the simulation data, and the parameters are adjusted on the verification set of the simulation data. The source model was established after complete model training and performance evaluation. After finishing the training of the source model, the parameters of each neuron in the neural network have been saved. The parameters will be copied to the target model for fine-tuning the experiment data. 20 epochs have been taken at fine-tuning the training process. In the case of training from scratch on experiment data, the training conditions are the same as the fine-tuning link of transfer learning, except for the initialization of the model.

The hyperparameter of TabNet is determined by our parameter adjustment work. The hyperparameter settings for the pre-training process and the fine-tuning process for a task are the same. We need to adjust the work according to the performance of the two-step training of a task. Table 1 shows the hyperparameter selection of the TabNet for each task. $N_{d}$ is the width of the decision prediction layer. The abbreviations in the table are described as follows. $N_{a}$ is the width of the attention embedding for each mask. Steps is the number of steps in the architecture. Lr is the learning rate.

The evaluation indexes of regression task accuracy are Pearson correlation coefficient (R), Mean Absolute Error (MAE) and root mean square error (RMSE). The evaluation index of the classification task is a function of accuracy.

## 3. Results

### 3.1. Data Analysis

The simulation datasets of five tasks were assembled as large as possible. The parameters of the alloy are referenced in the literature, and then the composition is adjusted randomly to generate a large-size simulation dataset. The experiment dataset of each task is collected from the published literature. The size of the experimental data is similar to the previous material properties prediction studies, which is in the range of 300–1000. In the experiment dataset, some elements such as C, Cr, Mn, etc occur in most samples, while Cu, Co, etc occur in much fewer samples. With unbalanced data distribution and small data sizes, it is difficult to build machine learning models using the experiment dataset.

**Figure 3:**
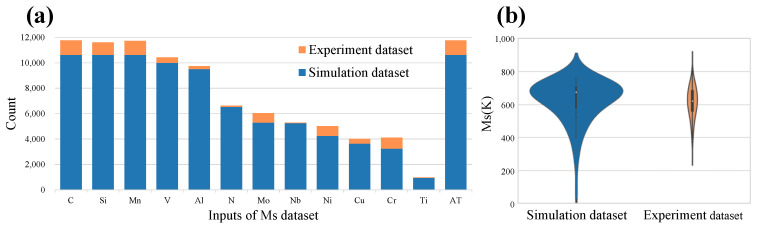
Statistics of Ms simulation dataset. (**a**) The occurrence counts of elements in the Ms simulation dataset and experiment dataset. (**b**) Violin plot of output variable of Ms simulation dataset and experiment dataset.

In Figure 3, we take the Ms simulation task as an example to show the distribution of input and output variables between simulation datasets and experiment datasets, respectively. Figure 3a shows the input elements of the dataset and their occurrence frequencies. The rightmost column is the austenitizing temperature (AT) of each alloy sample, and the rest is listed as the chemical elements contained in the data set. Except for the Ti element, the occurrences of other elements in the simulation dataset are all more than 3000 times, indicating that the input of the manually collected simulation dataset is widely distributed. Explain that the simulation data will completely contain the knowledge of the corresponding functions of the simulation software. The count of each element in the experimental dataset is significantly small, and the distribution of elements is very unbalanced. Figure 3b is the violin plot that shows the distribution of Ms values in the simulation dataset and the experiment dataset. The Ms values of the two datasets are concentrated around 600–800 K. In the simulation dataset, there are also some extreme values distributed at >900 K and 0–500 K. These extreme values are usually regarded as abnormal data that are not recorded in the experimental dataset. The statistical results show that the distribution of material experimental data is unbalanced and biased. The unbalanced and biased distribution makes it difficult to completely reflect the inherent laws of alloy materials. The simulation dataset is widely and evenly distributed, which will fully reflect the knowledge of the corresponding functions of the simulation software.

The similarity between the two kinds of data is a prerequisite for transfer learning. In the above, we showed the difference in the input and output distribution of the two datasets, respectively. However, the mapping between the input and output of the two datasets has a high degree of similarity. The high consistency proves the reliability of the simulation data, so we can extract the knowledge from the simulation data for knowledge transfer. We use maximum mean discrepancy (MMD) to show the similarity between the simulation data and the experiment dataset of the same task [34]. The equation of MMD is:

$$MMD\left(X,Y\right)={∥\frac{1}{n}\sum_{i=1}^{n_{1}}\phi \left(x_{i}\right)-\frac{1}{m}\sum_{j=1}^{n_{2}}\phi \left(x_{j}\right)∥}_{H}$$

where *X* and *Y* are two datasets, *n* and *m* are the sample size of *X* and *Y*, and ϕ() is the mapping between the input and output of a dataset. The MMD value between the simulation dataset and the experiment dataset of each task is counted in Table 2. The MMD values of each task are close to zero, which indicates that the simulated and experimental datasets of each task have similar mapping relationships. In fact, the distribution of data from two different sources cannot be exactly the same. For two different but related data distributions of the same task, we aim to take advantage of the similarities between the two datasets. And use the experimental data as the ground truth to make the prediction results of the model approach the experimental results.

### 3.2. Extract Thermodynamics Knowledge from Simulation Data

**Figure 4:**
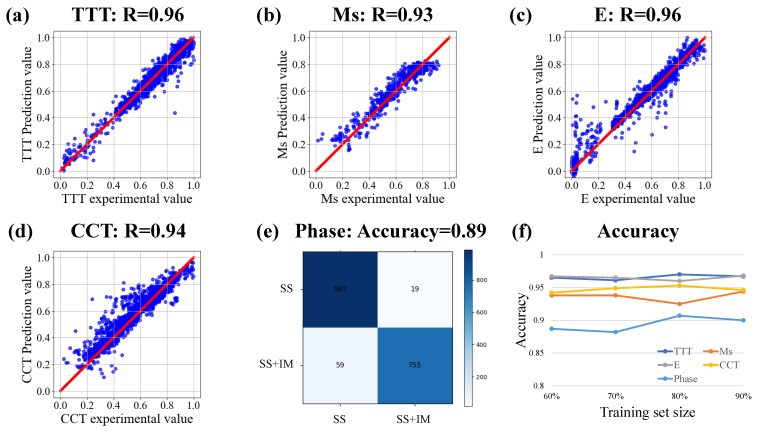
The accuracy analysis of source model. (**a**–**d**) Correlation analysis of TTT diagram, Ms, E, CCT diagram. (**e**) Confusion matrix of Phase. (**f**) Accuracy of each task under different training set sizes.

Figure 4 shows the accuracy of the source model for each task. It can be seen that the five tasks have achieved high accuracy in the learning of simulation data. The accuracy of the five tasks is about 0.9. The experimental results show that there is a high correlation between the predicted value of the source model and the simulation value. It is proved that the source model learns the rules in the simulation data. Figure 4f shows the accuracy of five tasks under different training set sizes. The horizontal axis shows the percentage of the training set in the total data in the current experiment, and the rest of the data is used as the test set. The split of the training set and test set is done randomly according to the split ratio. The accuracy shown in Figure 4f is the value obtained on the test set of each group. The results show that the network trained by each source model is stable in different sizes of training sets. It shows that the source model can stably learn the knowledge in the simulation dataset. The learned knowledge serves as the basis for subsequent transfer learning. The high accuracy indicates that the well-trained source model has learned the relationship between the input and output in the simulation data training set. It can accurately reproduce the simulation results of simulation software according to the input variables. In other words, each source model has learned the corresponding function of the simulation software to predict the corresponding properties of the alloy materials. In sum, we successfully extracted the material domain knowledge from the simulation data into the source model.

### 3.3. Transfer Learning of Experiment Data

The properties evaluation obtained from the experiment is the most real. In material properties prediction work, the ultimate goal is to make the prediction results as close as possible to the material experimental results. So the following machine learning model fine-tuning and the final performance evaluation take the experimental data as the ground truth. As concluded by the Data Analysis section, the experimental data for specific tasks in material science are often inadequate and unbalanced. These conditions hinder the application of machine learning. Previous studies on machine learning to predict material properties usually use descriptors selected by artificial feature engineering as input. However, the sophisticated input has increased the difficulty of the final application of the model. Because when using the model makes a prediction, it is necessary to match the form of the data to be predicted with the input and output of the model. In addition, due to the data size limitation, the prediction outside the range of the training data is not convincing. Under conditions of poor data quality, we aim to expand the scope of applications without increasing application restrictions. The specific strategy is to introduce domain knowledge for the machine learning model. In the previous section, we used simulation data for pre-training to extract domain knowledge into the source model. The domain knowledge will be used to guide the training of experimental data. In a neural network, the domain knowledge is saved as the parameters of each neuron. In the fine-tuning process, the parameters of each neuron in the source model are copied to the target model as initialization parameters. Then use the experiment dataset to retrain the target model. This ensures that the final model is learned according to the rules of the experimental data. Benefiting from domain knowledge, the target model can obtain higher accuracy and a wider range of applications than the randomly initialized model. In addition, the fine-tuning process after pre-training makes the predicted value of the target model closer to the experimental value than that of the simulation software.

**Figure 5:**
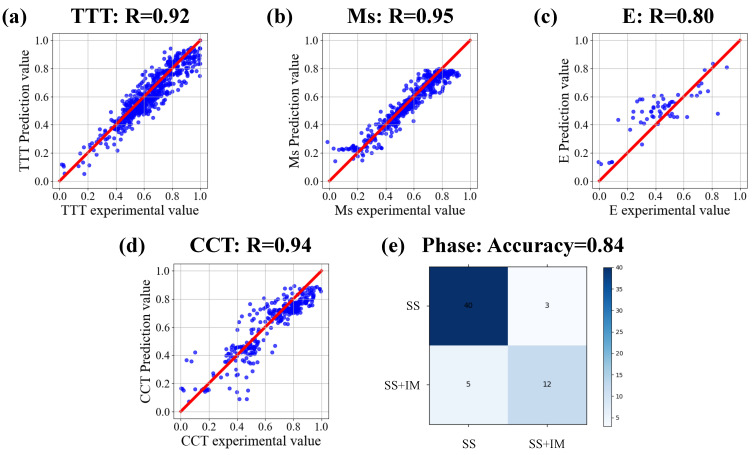
The target model accuracy of each task. Correlation analysis of target model predicted value and experimental value of (**a**) TTT diagram, (**b**) Ms, (**c**) E, (**d**) CCT diagram, (**e**) Phase.

Figure 5 shows the accuracy of the target model for each task. After fine-tuning, the target model containing domain knowledge can well fit the input and output of the experimental data. From the numerical results, it can be seen that the accuracy of the target model is high in the experimental data. The prediction accuracy of TTT, Ms, and CCT tasks is all above 0.9. E and phase tasks also reached more than 0.8. These result values are within the acceptable range. Such accuracy values indicate that the target model of each task has been successfully established. There is a high correlation between the predicted value and the experimental value. Without the help of feature engineering, the target model after transfer learning can also achieve high testing accuracy on the experimental data set with limited quality. Based on the above results, the proposed framework overcomes the inherent limitations of material experimental data sets and establishes an effective prediction model for multiple tasks.

**Figure 6:**
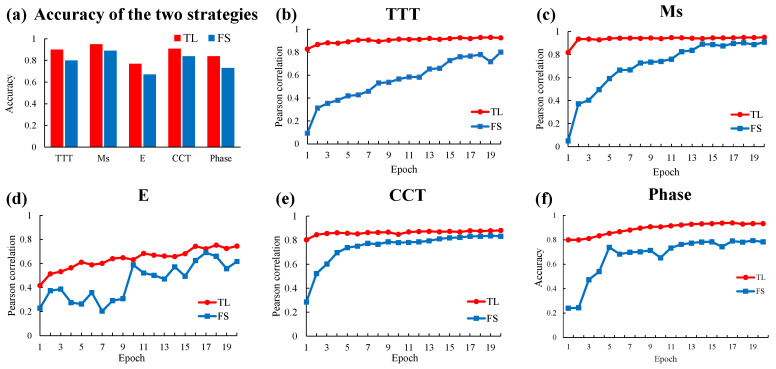
Effectiveness of transfer learning. (**a**) Comparison of the accuracy of the two strategies in the experimental data of each task. (**b**–**f**) Visualization of two strategies in the training process of each task.

To verify the effectiveness of transfer learning, we arranged a comparative experiment of deep learning training from scratch and transfer learning. The network architecture of the training from scratch strategy is consistent with that of transfer learning, while the network parameters are initialized randomly. The experiment was designed to determine whether the domain knowledge extracted by the source model could promote the learning of experimental data. In this experiment, the experiment group experiment was set as the transfer learning on the experimental datasets. The control group experiment was set as training from scratch on the experimental datasets for all five tasks. The difference between the control group and the experimental group lies in whether the network has domain knowledge. Through comparative experiments to verify whether domain knowledge can play a promoting role in the training of experimental data. The comparison result of the test accuracy of the two strategies on each task is shown in Figure 6a. We can conclude that all five tasks achieved higher accuracy in the transfer learning strategy. The accuracy of the experimental group was higher than that of the control group. The transfer learning strategy has more accuracy advantages under the same training conditions. The experimental results prove the effectiveness of transfer learning and the promoting effect of domain knowledge on the learning of experimental data.

Figure 6b–f are visualizations of the training process for each task. The rising trend of the curve in the figure indicates that the accuracy of the test set is gradually improved with the increase of the training cycle. By comparing the rising trend of accuracy between the experimental group and the control group, it can be found that the overall height of the curve of the experimental group is higher than that of the control group. This means that the accuracy of the experimental group is always higher than that of the control group in the same training round. We can also observe that the curve of the experimental group changes from rising to flat faster than that of the control group. In other words, transfer learning strategies can achieve convergence faster. At the end of the observation curve, which is the last epoch, the final convergence result of the experimental group was higher. This proves that the transfer learning strategy can achieve higher accuracy under the same computational cost. At the starting point of the observation curve, that is, in the earliest era, the accuracy of the experimental group was significantly higher than that of the control group. It is proved that the domain knowledge acquired through pre-training plays a role so that the target model can start training with better parameters. In short, transfer learning strategies allow the model to have prior domain knowledge in its initial state. The domain knowledge can promote the process of deep neural network learning experimental data. It makes the learning process faster, reduces the computational cost, makes the final effect of learning more accurate, and improves the prediction effect of the model. The experimental results verify the effectiveness of the transfer learning strategies used in our proposed framework. The transfer learning strategy can perform better on the experimental dataset because the model has acquired reliable domain knowledge and then conducted further training on the experimental data. The comparison result further demonstrates that the domain knowledge contained in the machine learning model can effectively improve the material properties prediction performance of the machine learning method under the current data situation.

**Figure 7:**
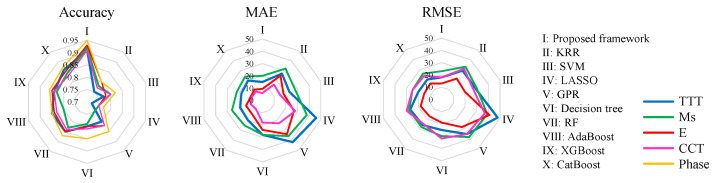
Comparison of testing performance of conventional machine learning methods and proposed framework.

To further prove the accuracy advantage of the proposed framework, we also employed another set of comparative experiments with conventional machine learning methods. Nine commonly used machine learning methods in material property prediction were selected, namely kernel ridge regression(KRR) [35], support vector machine(SVM) [36], LASSO [28], gaussian process regression (GPR) [29], decision tree, Random Forest (RF) [30], adaptive boosting(AdaBoost) [37], extreme gradient boosting(XGBoost) [38], and categorical boosting(CatBoost) [39]. The procedures of the above methods were run in Python, with the help of Scikit-learn and other libraries. Based on the theoretical analysis and experimental verification in the previous sections, the proposed framework should be able to achieve higher accuracy than conventional machine learning methods. The radar chart in Figure 7 shows the comparison of different evaluation indicators between the proposed framework and the conventional machine learning method. The evaluation indexes of regression tasks are accuracy, MAE, and RMSE, and the evaluation index of the classification task is accuracy. It indicates that the proposed framework achieves higher accuracy than all other conventional methods. The comparison result proves that the proposed framework significantly improves alloy properties prediction performance when using regular material datasets with simple inputs. Due to the size and quality of the experiment data, the relationship between the composition and properties of the alloy can not be accurately reflected. This restricts machine learning methods from making high-accuracy predictions. The proposed framework uses domain knowledge to supplement the laws of the experiment data, so it makes predictions with higher accuracy. The above results clearly demonstrate that the proposed framework is a productive method to cope with data limitations.

In this work, another goal is to eliminate the calculation errors caused by assumptions and idealized premises in the simulation software. The simulation software calculates the properties of the material according to the built-in formula. There are certain idealized conditions in these formulas. This will inevitably lead to discrepancies between the calculated results and the real situation. In most cases, these errors will be accepted when using simulation software to calculate the properties. However, when we carefully compare the simulation results with the experimental results, we will find that the error is actually relatively large. So, from another point of view, we hope to eliminate these errors through a small amount of experimental data. Our proposed framework is first pre-trained with simulation data and then fine-tuned with real data, so that the network learns the functions of the simulation software and then approaches the real situation. Finally, a model which is closer to the real data than the simulation software is obtained. Table 3 shows the performance comparison between the proposed framework and the simulation software. We can see that there is an overall improvement in the accuracy of our framework. A more reliable prediction method is established than the simulation software.

### 3.4. Model Validation

**Figure 8:**
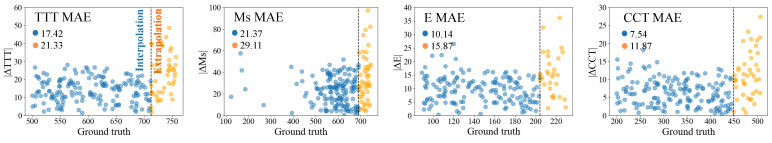
Absolute differences in predicted and experiment value against the experiment value range for the validation data.

Besides achieving high accuracy in the prepared dataset, reliable extrapolation ability is also an important part of the prediction ability of the machine learning model. Thus, we carried out the extrapolation ability verification experiment to show the prediction effect on unseen data. The unseen data is set to the experiment data outside the scope of the training set. We use the forward cross-validation method to verify the extrapolation performance of the model, which divides the experiment data according to the range of target values [40]. In the extrapolation verification experiment, the samples with the top 20% of the output value are taken as the extrapolation data, the remaining data is divided into interpolation data. After dividing the data, two disjoint regions are formed to perform the extrapolation verification experiment. Figure 8 shows the result of extrapolation verification for four tasks. When the prediction data is outside the scope of the training data, there is no huge loss in the prediction ability of the model. The experiment is based on the premise that the test dataset and the training dataset are completely disjoint, which proves that the framework can train a model with good prediction ability for completely unseen data. These results demonstrate that the proposed framework can significantly enhance models’ accuracy in extrapolation tasks critical in new materials discovery.

**Figure 9:**
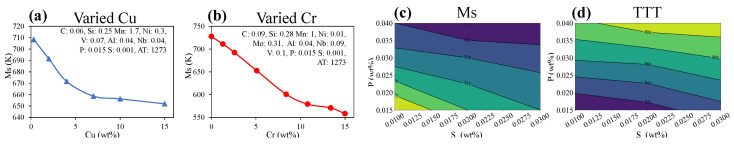
The influence of elements on properties in framework prediction. (**a**,**b**) The influence of single element on Ms. (**c**,**d**) Contour map.

The correctness and interpretability of the prediction process are also important factors in evaluating the proposed framework. The machine learning model should be verified whether the way it makes predictions is consistent with the principle in reality. We set up experiments to prove that the prediction process of the model is the same as the principle in reality, instead of obtaining the correct result with an unexplainable black box. The proposed framework learns the relationship between alloy composition and properties. According to metallurgical experience, the degree of influence of different elements on material properties is variable. The following experiment proves that the influence trend of element content on material properties is consistent with the actual metallurgical experience. Figure 9a,b show the effects of Cu and Cr elements on the martensite start temperature in the alloy, respectively. The other elements of the alloy and the austenitizing temperature are shown in the upper right corner of the corresponding figure. With the increase of Cu and Cr element content, the martensite starting temperature of the alloy shows a downward trend under the same austenitizing temperature (1273 K). The downward trend becomes flat when the Cu element content rises to about 6wt%, but there is no obvious trend change when the Cr element content increases. The following experiment shows the influence of trace elements P and S on the properties of the alloy. The interaction of P and S elements with other elements of the alloy causes changes in the alloy properties. Figure 9c,d shows the prediction results through high-dimensional contour maps, showing that the influence of alloying elements on properties is nonlinear, which is consistent with the facts. It has been confirmed that P and S elements interact with other elements to affect the properties of the alloy in a complex manner. The traditional empirical formula or simulation simplifies the influence of alloy elements on linear relationships. The result shows that the alloy property prediction function of the framework is more advanced. The prediction results of Co-Cr-Fe high entropy alloys are given in Table 4, and the experimental results are directly compared with the predicted results. The results show that our framework can make correct predictions in most cases, but it may make mistakes at the critical point when a new phase is generated or when a phase disappears. The prediction made by the model established following the proposed framework is consistent with the metallurgical experience. Compared with metallurgical experience, the correctness and interpretability of the framework are verified.

### 3.5. Transfer Learning of Unseen Properties

We have demonstrated that the proposed framework achieves considerable performance on experimental datasets using transfer learning strategies. Previous experiments were carried out on tasks that included both the simulation dataset and the experiment dataset. However, the functions of simulation software are limited. Not all material properties can generate a large number of simulation data through the corresponding simulation software. In addition, collecting a large amount of simulation data is also tedious work. In this section, we aim to prove that the framework can be applied to unseen property predictions. The new property mentioned above is the task that there is no direct corresponding simulation dataset, but only the experimental dataset. From the point of transfer learning, the new material property prediction task and the existing tasks have different output domains and highly correlated (or overlapping) input domains. Common elements in those alloys are similar. And there is an inseparable relationship between the different properties of an alloy. So, we believe that the extracted features of the trained deep neural network are instructive for predicting new material properties.

We chose glass-forming ability(GFA) as the new material property. GFA data sets are experimental data collected from published literature. The input form of the GFA data set is the same as the previous task, which is the composition of the alloy. The output of the GFA dataset is the specific value of glass-forming ability.

In deep learning, the parameters of the intermediate layers of the network are called features. These characteristics contain knowledge of the correspondence between alloying element composition and alloy properties. Because of the existence of correlations between different properties of alloys, we hope to reuse this knowledge to aid in the training of unseen properties. We use the five collected simulation data sets as domain knowledge to carry out the transfer learning on experiment data of the new property. The intermediate layer initialization parameters of the network use pre-trained parameters, and the final classification layer is randomly initialized. These five transfer tasks are then aggregated using the concepts of the ensemble in machine learning to get the best results on new task predictions. Table 5 shows the performance evaluation scores of our transfer learning model on the new property dataset. Our model does not need any human feature engineering, and after training and testing, it can achieve an accuracy of about 87%, while ensuring a high precision and recall value. Domain knowledge is embedded in the middle layer of the network and reflected in the extracted features, but this knowledge can not be obtained by ab initio training. The proposed framework is an upgrade of traditional machine learning based on artificial feature engineering and could provide effective guidance for new material property prediction.

## 4. Discussion

In the design process of new alloys, it is one of the crucial steps to determine the alloy composition in the huge composition space. The content of the composition directly affects the properties of the designed alloy. Therefore, the appropriate composition needs to be selected in the alloy design process, so that the target alloy can meet the requirements of its application environment. The Material experiment is the most accurate method to measure the properties of newly designed alloys. However, it is limited by the highly time-consuming and high-cost effects caused by the complexity of material experiments. And the error in the experimental process leads to the need for repeated experiments to ensure the accuracy of the results, which further increases the cost and time consumption of the design process. To reduce the cost while obtaining accurate alloy properties, it has become an urgent need to establish a prediction model of the composition-property relationship. We propose a prediction framework of alloy properties based on deep transfer learning, which can accurately predict multiple properties of the alloy. The experimental results show that the framework can accurately predict the target properties of the designed alloys, and the framework is a general method that can be applied to many kinds of alloys and properties.

The major strategy of our framework is to couple domain knowledge with machine learning models through transfer learning. With the help of transfer learning, the simulation data can be used as a supplement to the experiment data in the form of domain knowledge. Machine learning models supplemented by domain knowledge can get higher accuracy and more reliable prediction results. The properties of the alloy predicted by the model are very close to the experimental values. And it achieves higher accuracy than conventional machine learning methods. First of all, through the data distribution experiment, we prove that there is a strong consistency between the simulation data and the experimental data. However, due to the introduction of errors in the idealized assumptions in the thermodynamic formulas embedded in the simulation software, there is still a deviation between the simulation data and the experiment data. We aim to use the strong consistency between two kinds of data and use simulation data to help the learning of experimental data. Because the simulation data are calculated by the thermodynamic formula embedded in the simulation software, simulation data can be incorporated into deep neural networks in the form of reliable domain knowledge. Verified by experiments, we have successfully extracted the domain knowledge from the simulation data. In the process of knowledge transfer, the network parameters of the source model trained by the simulation data are copied as the initialization parameters of the target model of experimental data. The initialized network parameters promote the learning of small-scale experimental data. Several comparative experiments have been carried out to verify the accuracy of the proposed framework. Comparative experiments show that our framework can achieve higher accuracy than conventional models when simple features (composition and treatment process) are used. Finally, we demonstrate the correctness and interpretability of the proposed framework to prove that the model built by the framework is not a black box. In particular, we apply the proposed framework to five different alloy properties prediction tasks. Similar experiment results are obtained on five tasks, which proves the generalization of the framework.

## 5. Conclusions

In sum, deep neural networks combine domain knowledge in the form of network parameters through learning simulation data. Domain knowledge is applied to the learning of experimental data through the transmission of network parameters. The framework is beneficial to reduce the negative impact of limited data collection on machine learning methods. The specific contributions are described below. The existence of domain knowledge assists in increasing the predictive reliability of machine learning. Compared with experimental data, large-scale simulation datasets with a wider range of data also play a role in model training, enabling models to make reliable predictions outside the scope of experimental datasets. In addition, the proposed framework uses simple element content and a small number of process parameters as the input of the neural network. Compared with the traditional machine learning method, this framework does not need to construct complicated descriptors to improve precision, so the complexity of using the framework is lower. This means that the proposed framework can be adapted to the rawer data. And the proposed framework is a general method. As long as the data of related tasks are collected and simple processing is performed, the machine learning model of the corresponding task can be trained according to the framework process.

## Figures and Tables

**Table 1:** Hyperparameters of each task model.

Hyperparameter	TTT	Ms	E	CCT	Phase
$N_{d}$	8	8	16	8	16
$N_{a}$	8	8	16	8	16
steps	2	2	2	2	2
lr	0.01	0.01	0.01	0.1	0.1
optimizer	Adam	Adam	Adam	Adam	Adam

**Table 2:** MMD values between two kinds of data for five tasks.

Task	TTT	Ms	E	CCT	Phase
MMD	0.0199	0.4793	2.1783	0.5038	2.1739

**Table 3:** Accuracy comparison between the proposed framework and the simulation software.

Prediction Model	TTT	Ms	E	CCT	Phase
Jmatpro	0.85	0.72	0.65	0.71	-
Thermo-calc	0.81	-	-	0.74	64%
Our	0.92	0.95	0.80	0.94	84%

**Table 4:** Verification of alloy phase prediction results of Co-Cr-Fe system high entropy alloys.

No.	Co/at%	Cr/at%	Fe/at%	Ni/at%	Si/at%	Nb/at%	Prediction Result	Literature Result
1	25.23	20.23	30.21	24.32	0	0	SS	SS
2	24.68	21.30	28.46	23.84	0	1.73	SS + IM	SS + IM
3	25.11	21.11	27.49	24.47	0	1.82	SS + IM	SS + IM
4	25.10	20.46	26.39	24.94	0	3.10	SS + IM	SS + IM
5	23.34	21.68	27.68	24.15	0	3.16	SS + IM	SS + IM
6	23.07	22.05	28.43	22.60	0	3.85	SS + IM	SS + IM
7	31.81	0	35.51	32.68	0	0	SS	SS
8	30.52	0	30.89	30.27	8.31	0	SS	SS
9	28.16	0	27.73	27.28	16.83	0	SS	SS + IM
10	25.72	0	26.31	26.06	21.91	0	SS + IM	SS + IM

**Table 5:** The scores of transfer learning on high-entropy alloys dataset.

Knowledge Source	Accuracy	Precision	Recall	F1 Score
TTT	0.807	-	-	-
Ms	0.752	-	-	-
CCT	0.812	-	-	-
E	0.738	-	-	-
Phase	0.821	-	-	-
Ensemble	0.872	0.878	0.871	0.881
None	0.684	0.679	0.682	0.660

## Data Availability

The data presented in this study are available in the Supplementary Materials.

## Supplementary Materials

The following are available at: https://www.mdpi.com/article/10.3390/ma15217442/s1.
